# Invasive pericardial and pulmonary aspergillosis by uncommon *Aspergillus* species in anti-interferon-γ autoantibody-associated immunodeficiency: a case report

**DOI:** 10.3389/fmed.2026.1760017

**Published:** 2026-04-16

**Authors:** Junyuan Tang, Zhijuan Luo, Yunhong Li, Wenbo Jiang, Yunying Weng, Guixin Zhang, Cheng Li, Yuhong Liu, Xuejiao Sun, Lin Chen

**Affiliations:** Department of Respiratory and Critical Care Medicine, Liuzhou People's Hospital Affiliated to Guangxi Medical University (Key Laboratory of Asthma and Chronic Obstructive Pulmonary Disease Diagnosis, Treatment and Research, Liuzhou), Liuzhou, Guangxi, China

**Keywords:** anti-interferon-γ autoantibody, *Aspergillus siamensis*, *Aspergillus udagawae*, granulomatous inflammation, immunodeficiency syndrome, invasive aspergillosis, metagenomic next-generation sequencing, multiple organ failure

## Abstract

This case report describes a 51-year-old female patient who presented with dyspnea and was diagnosed with invasive aspergillosis affecting the pericardium and lungs, secondary to immunodeficiency syndrome caused by anti-interferon-γ autoantibodies. Diagnosis was established by pericardial tissue metagenomic next-generation sequencing (mNGS) identifying *Aspergillus udagawae* and serum anti-interferon-γ autoantibody testing (titer 1:2,500). Despite sequential antifungal therapy with voriconazole, isavuconazole, and amphotericin B, the patient developed progressive multifocal infection, including an abdominal wall abscess and mediastinal infection caused by *Aspergillus siamensis*, and ultimately died of multiple organ failure. This case highlights the diagnostic challenges and poor prognosis associated with this rare immunodeficiency syndrome and emphasizes the importance of early recognition, precise pathogen identification, and consideration of immunomodulatory therapy.

## 1 Introduction

Anti-interferon-γ autoantibody (AIGA)-associated immunodeficiency syndrome is a newly identified adult-onset immunodeficiency first reported in 2004 (1, 2). Patients with this syndrome often present with severe, complex opportunistic infections, which are associated with a poor prognosis. Common pathogens include non-tuberculous mycobacteria, *Salmonella typhi*, *Burkholderia* species, *Cryptococcus neoformans*, and others. Recurrent infections frequently lead to septic shock, multiple organ failure, and even death (3, 4). This paper aims to enhance clinicians’ understanding of this disease by providing a detailed analysis of a case of invasive aspergillosis affecting the pericardium and lungs as a secondary complication of this syndrome. It reviews the clinical data, diagnostic pathway, treatment course, and key reflections to promote early, accurate identification and effective intervention.

## 2 Clinical data

### 2.1 Initial presentation

A 51-year-old female patient was admitted to the Department of Respiratory and Critical Care Medicine on November 12, 2024, due to dyspnea lasting for 5 days. The patient developed dyspnea without apparent precipitating factors, which worsened with exertion. She also presented with cough, sputum production, abdominal distension, fatigue, poor appetite, nausea, and occasional palpitations. Physical examination revealed a temperature of 36.4 °C (97.5 °F), pulse rate of 112 beats per minute, respiratory rate of 20 breaths per minute, and blood pressure of 99/71 mmHg. Her body mass index (BMI) was 20.8. The patient was alert and oriented. No rales were detected in either lung. Her heart rhythm was regular, and no cardiac murmurs or pericardial friction rub was heard over any valve area. Mild edema was observed in both lower extremities.

### 2.2 Laboratory and imaging findings

**Figure 1:**
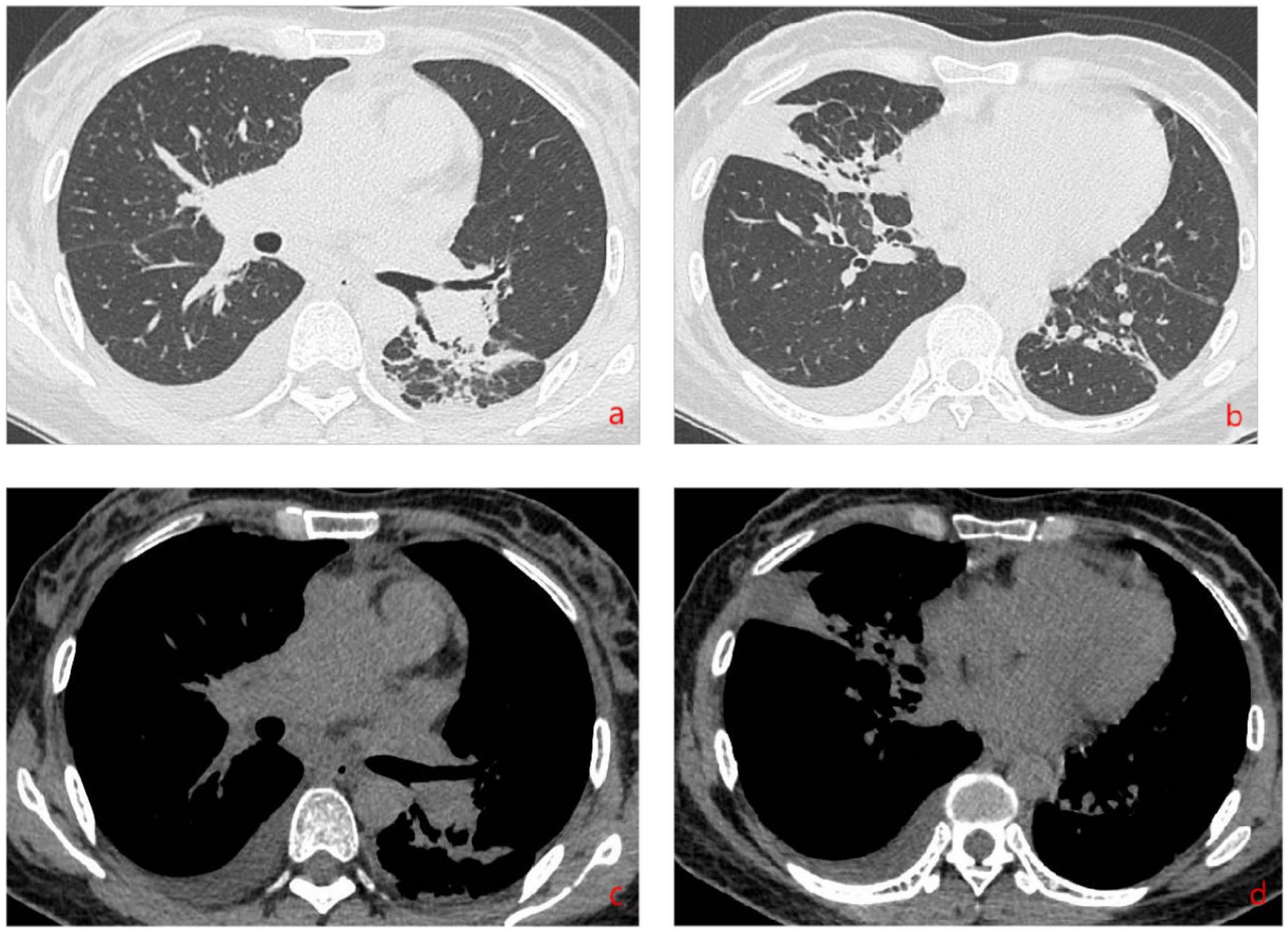
Chest CT on admission (November 12, 2024). **(a–d)** Show signs of *Aspergillus* infection in both lungs, small amount of pericardial effusion, multiple enlarged lymph nodes in both hilar regions and mediastinum, and small amounts of pleural effusion bilaterally.

Complete blood count: white blood cell count 5.23 × 10^9^/L, hemoglobin 95 g/L, absolute lymphocyte count 0.62, absolute neutrophil count 4.05. CRP 79.68 mg/L. IL-6 39.540 pg/mL. Albumin 29.9 g/L. Antinuclear antibody 260 AU/mL, anti-Sm antibody IgG 110 AU/mL. Lymphocyte subset analysis: helper/inducer T lymphocyte percentage 20.1%, B lymphocyte percentage 35.5%, helper/suppressor T lymphocyte ratio 0.63%, absolute total T lymphocyte count 315 cells/μL, absolute helper/inducer T lymphocyte count 115 cells/μL, absolute suppressor/cytotoxic T lymphocyte count 182 cells/μL, absolute lymphocyte count 573 cells/μL. Sputum nucleic acid test: positive for *Aspergillus* species (Ct value 21.9). Chest CT (Figure 1) showed consolidation and exudative changes in the right middle lobe; small pericardial effusion; multiple enlarged lymph nodes in both hilar regions and mediastinum; small bilateral pleural effusions. The admission diagnosis was community-acquired pneumonia (CURB-65 score: 0), pulmonary aspergillosis, chronic gastritis, mild anemia.

### 2.3 Initial treatment and disease progression

**Figure 2:**
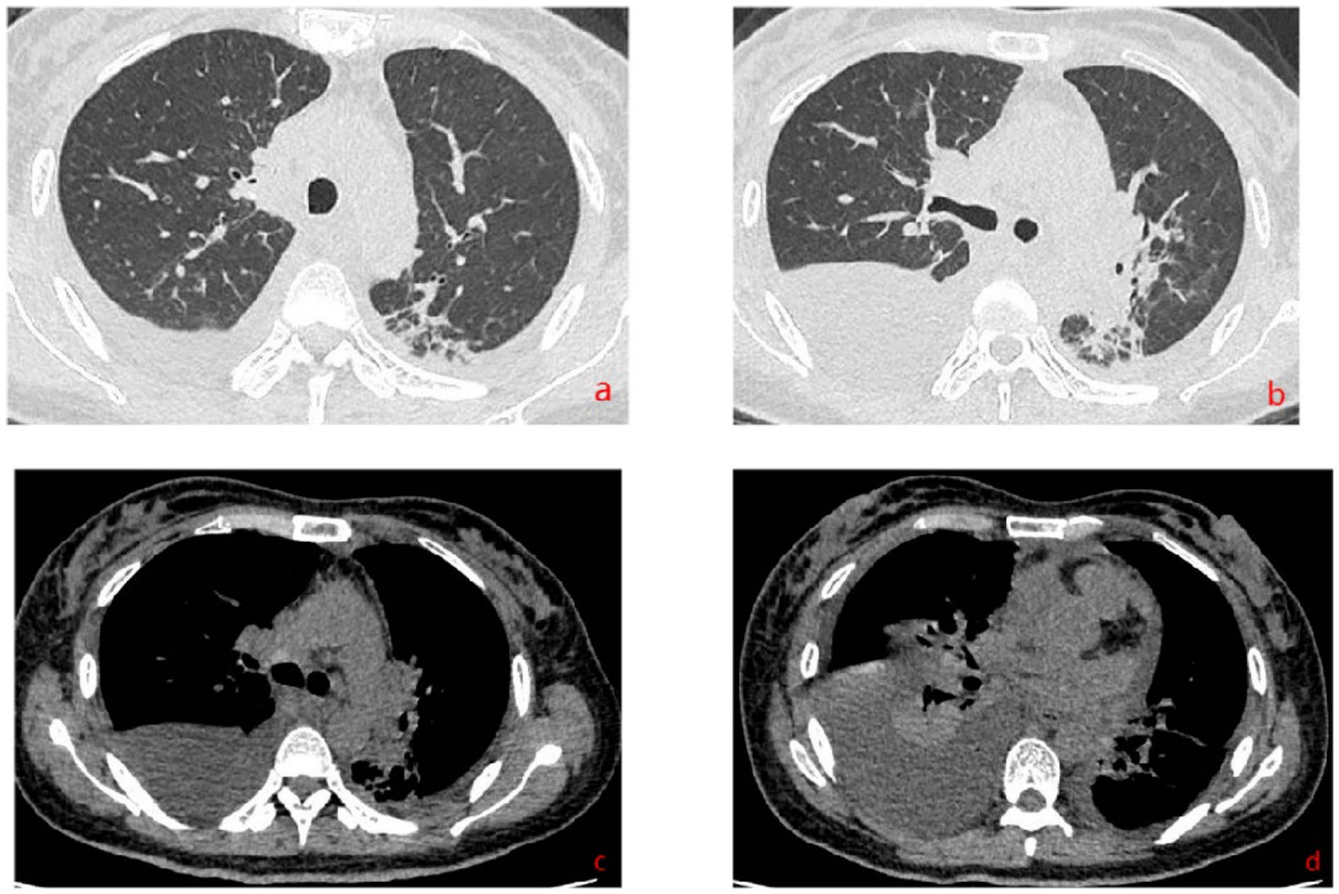
Pulmonary artery CT (November 15, 2024). **(a–d)** Show no signs of pulmonary artery embolism, uneven thickening of the pericardium and mediastinal soft tissues, and pulmonary vein stenosis. Findings are suggestive of inflammatory changes secondary to fibrinous mediastinitis; metastatic tumors cannot be excluded. Pulmonary lesions and pleural effusion are increased compared to previous findings, with a small amount of pericardial effusion.

**Figure 3:**
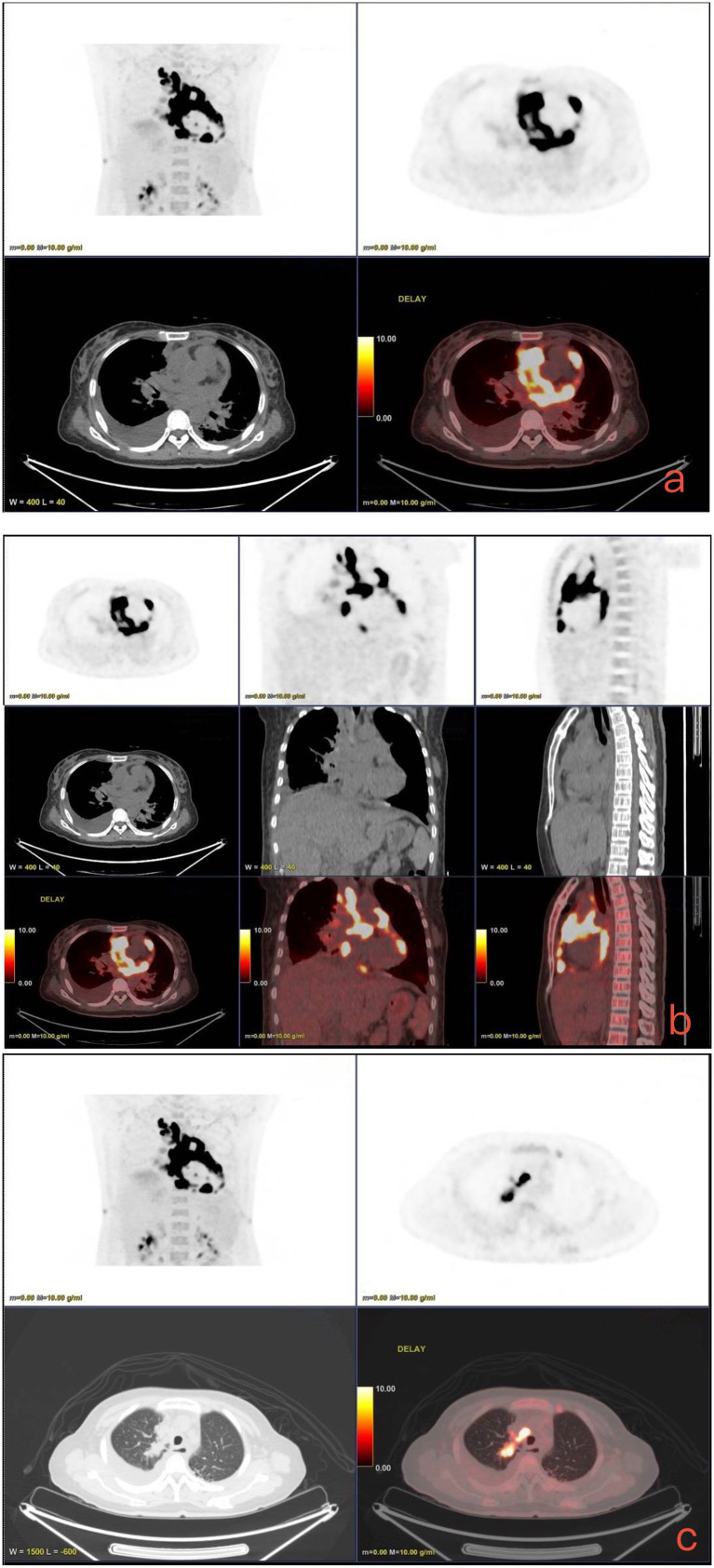
PET/CT (November 18, 2024). **(a–c)** Show uneven enhancement of the pericardium and mediastinal soft tissues with increased glucose metabolism, highly suggestive of a specific infectious lesion.

**Figure 4:**
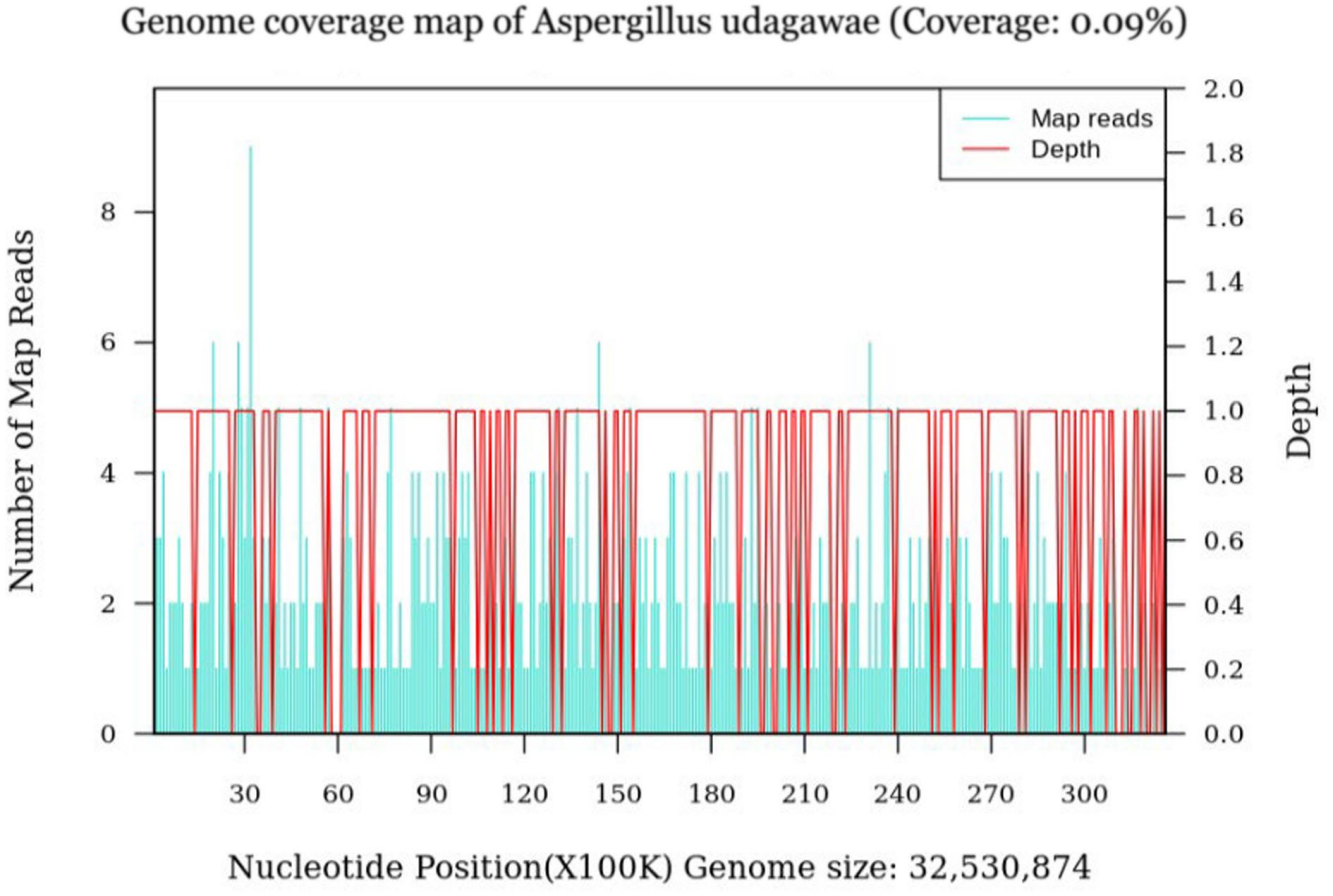
mNGS (December 1, 2024): Pericardial tissue from the patient reveals *Aspergillus udagawae*.

**Figure 5:**
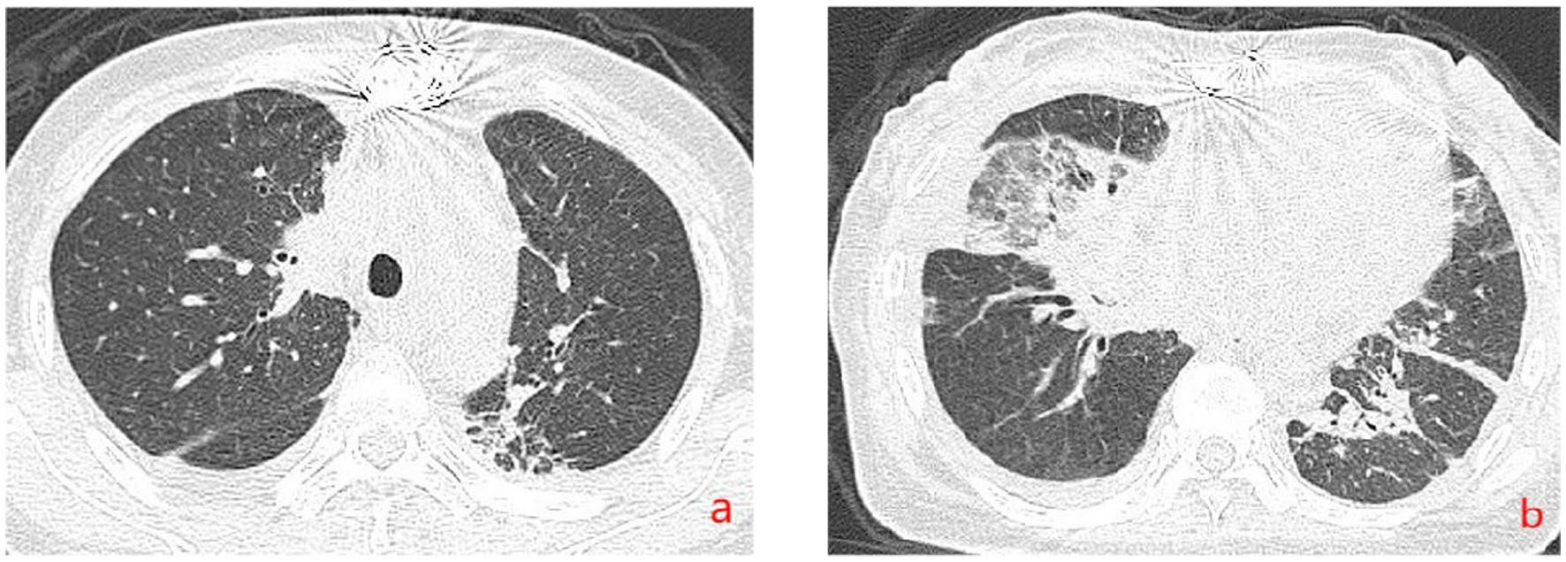
Chest CT (December 29, 2024). **(a,b)** demonstrate post-pericardial stripping changes. Mediastinal exudate and effusion are decreased compared to previous findings. Uneven thickening of the pericardium and mediastinal soft tissues is reduced. Lesions in the right lung are increased compared to previous findings.

The patient’s dyspnea improved after treatment with oral voriconazole (200 mg bid) for antifungal therapy, intravenous piperacillin-tazobactam (4.5 g q8h) for anti-infection, plus antispasmodics, bronchodilators, gastric protection, pleural drainage, and low-flow oxygen therapy. Contrast-enhanced chest CT (Figure 2) revealed uneven pericardial thickening and uneven mediastinal soft tissue thickening, suggesting possible secondary fibrinous mediastinitis; metastatic tumors could not be ruled out. Pulmonary lesions had increased compared to previous imaging. PET/CT (Figure 3) indicated uneven thickening with increased glucose metabolism in the pericardium and mediastinal soft tissues, suggesting specific infectious lesions, possibly pericardial tuberculosis. On November 29, 2024, open pericardiectomy was performed. Postoperative diagnosis included constrictive pericarditis (likely tuberculosis), pulmonary vein stenosis (right lung), pulmonary aspergillosis, and chronic gastritis. Postoperative management included endotracheal intubation for assisted ventilation, anti-tuberculosis therapy (Isoniazid 0.75 g qd + Pyrazinamide 0.5 g tid + Moxifloxacin 0.4 g qd + Linezolid 0.6 g q12h), antifungal therapy (voriconazole 200 mg q12h), and nutritional support. Pericardial pathology identified fungal elements consistent with *Aspergillus* (Figure I in Table 1). Pericardial tissue metagenomic next-generation sequencing (mNGS) indicated *Aspergillus udagawae* infection (Figure 4). Anti-tuberculosis drugs were discontinued, and voriconazole antifungal therapy was continued. The patient’s condition gradually improved, with extubation and discontinuation of ventilator support. On December 19, 2024, voriconazole blood concentration was 6.38 μg/mL, and the dose was reduced to 150 mg. Repeat chest CT on December 27, 2024 (Figure 5) showed increased pulmonary lesions, considered related to postoperative exudative effects and suboptimal antifungal response. Antifungal therapy was switched to oral esavuconazole, while piperacillin-tazobactam antibacterial therapy was continued. One week after taking isavuconazole, the patient developed adverse reactions including lower limb edema, fatigue, poor appetite, and poor mental status. On January 3, 2025, treatment was switched to amphotericin B, with the dose gradually increased to 25 mg/day. The patient developed drug-induced liver injury, and glutathione was added for hepatoprotection. By January 13, 2025, the patient’s symptoms improved, and she was discharged.

**Table 1:** Summary of multiple routine pathology examination reports for patient from 2019 to 2024.

Time	Specimen	Pathological description	Pathology images	Pulmonary Imaging
May 22, 2019	Transbronchial lung biopsy (transbronchial lung biopsy, TBLB): Right lower lobe, dorsal segment	Chronic mucosal inflammation. Immunohistochemistry: CK expression normal, Ki-67 (−). Special staining results: PAS (−), hexamine silver staining (−).	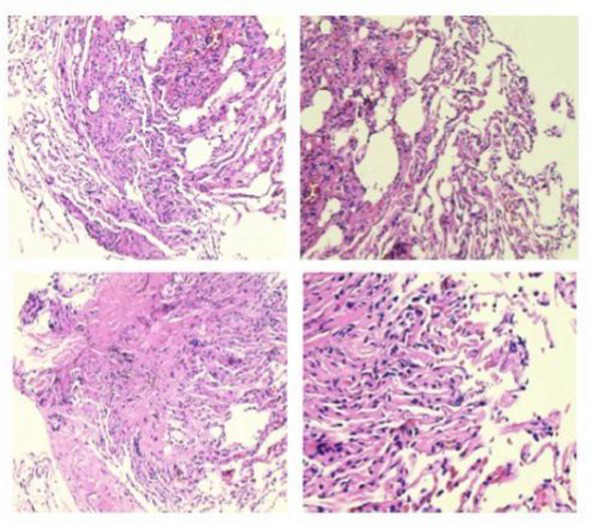 A	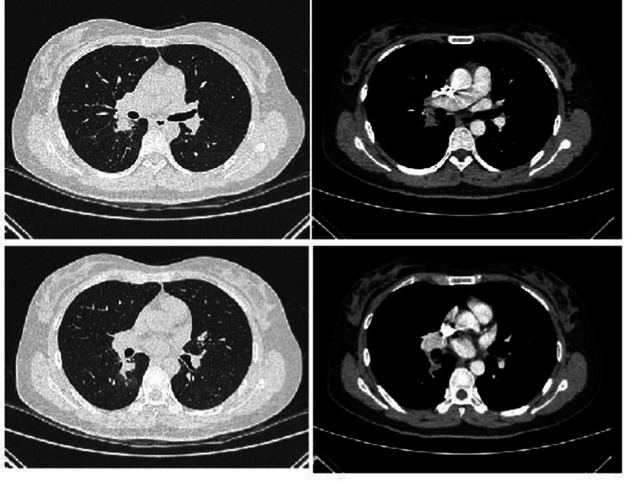 B
October 29, 2020	Percutaneous lung biopsy: Right upper lobe	Granulomatous inflammation; tuberculosis cannot be excluded. Please consult with clinical and other examination findings. Immunohistochemistry: CK epithelial (+), CD68 histiocyte (+). Special stains: PAS (−), acid-fast (−).	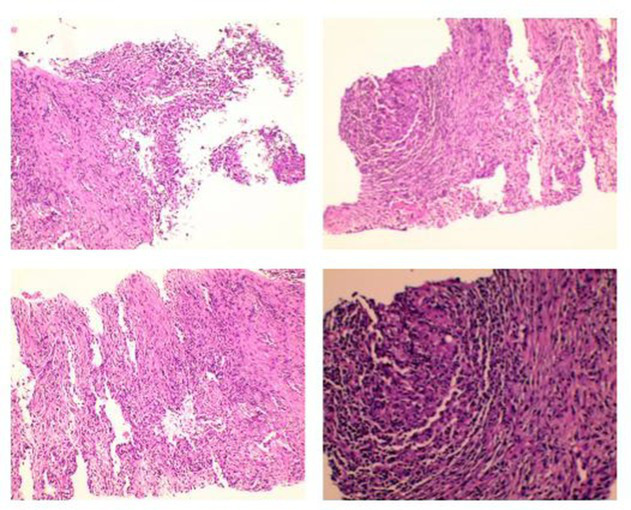 C	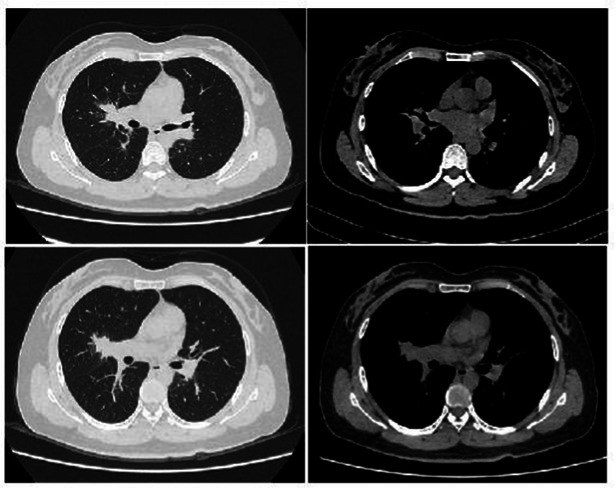 D
May 26, 2023	Debridement of left hand dorsum and right calf: Ulcer on left hand dorsum, ulcer on right calf	Granulomatous inflammation with adjacent abscess formation. Multinucleated giant cell reaction is observed. Tuberculosis could not be excluded; consult with tuberculosis specialists and perform relevant tests. Immunohistochemistry results: Specimen 2: CK positive in epithelial cells (+) CD68 positive in histiocytes (+) Special stains: Specimen 2: PAS stain shows no abnormalities, Hexamine silver stain (−), Acid-fast stain (−).	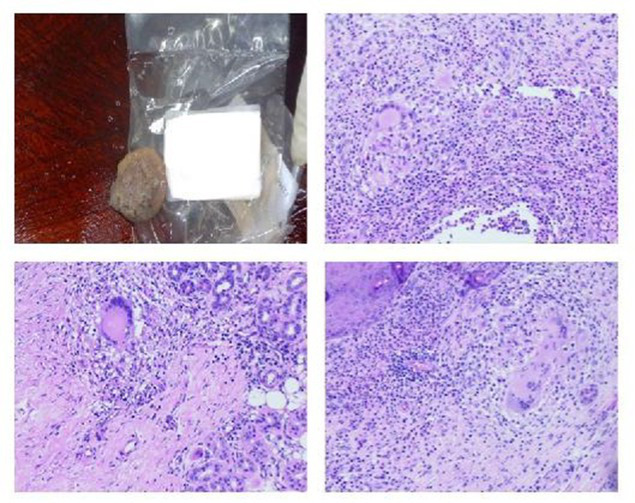 E	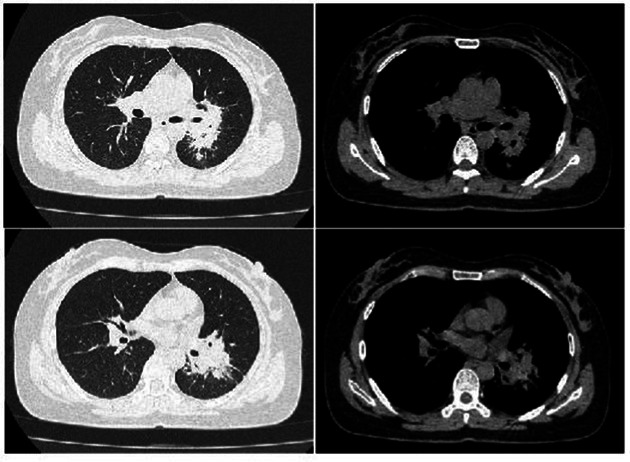 F
August 19, 2024	Bronchoscopic biopsy: Mass in the right main bronchus	Granulomatous inflammation with abscess formation. Immunohistochemistry results: CK, TTF-1, p63 (normal mucosal expression), CD68 (histocyte+), CD38 (plasma cell+). Special staining results: PAS (−), acid-fast staining (−), hexamine silver staining (−).	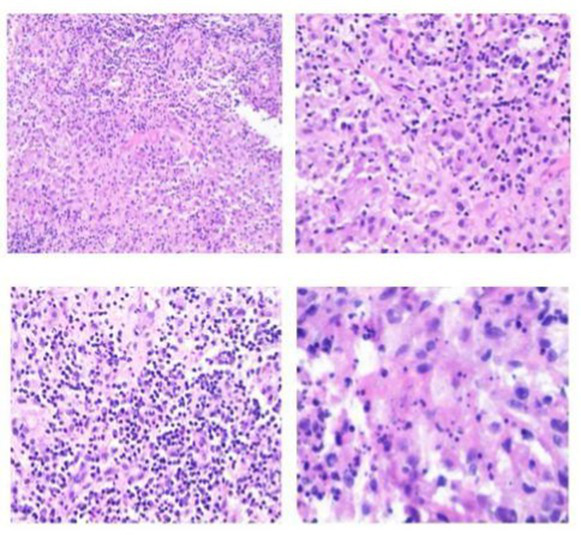 G	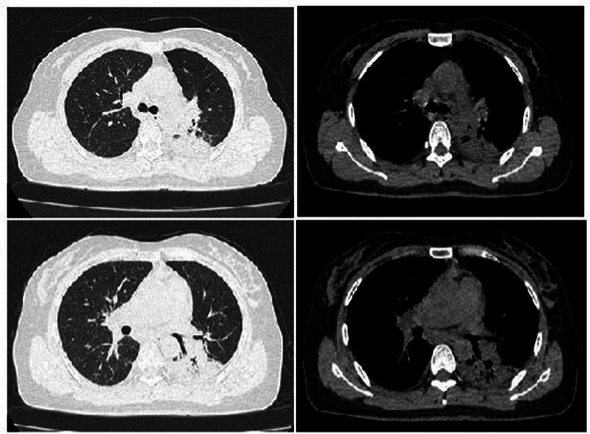 H
November 29, 2024	Open thoracotomy with pericardial resection: Mediastinal mass, pericardial tissue	The submitted tissue shows hyperplasia with degeneration in some areas, accompanied by extensive infiltration of lymphocytes, plasma cells, and neutrophils, necrosis, and abscess formation. In certain regions, histiocyte aggregation and multinucleated giant cell reactions are observed, along with granuloma formation. Fungal components are visible in small areas, morphologically consistent with *Aspergillus*. Immunohistochemistry: CK (+) in mesothelial cells; CD68 (+) in histiocytes. Special stains: PAS stain (+).	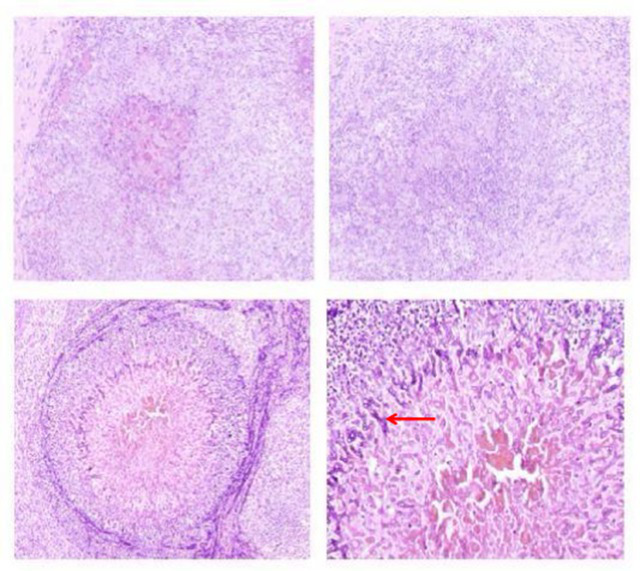 I	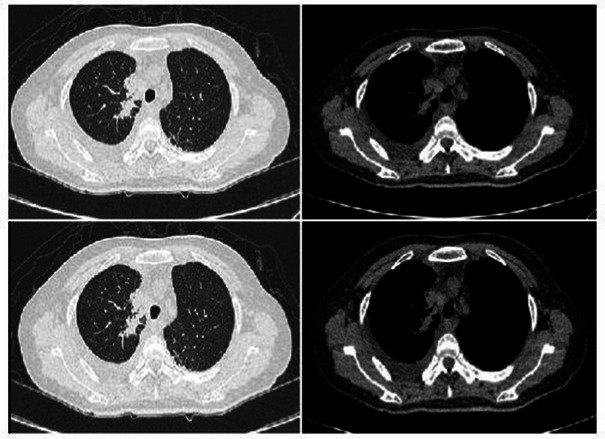 J

### 2.4 Readmission and final outcome

On January 23, 2025, the patient was readmitted due to substernal pain. Investigations revealed an abdominal wall abscess and mediastinal infection. The patient underwent abscess incision and drainage followed by abdominal wound debridement. Drainage fluid culture grew *Aspergillus siamensis*. Treatment included mechanical ventilation, anti-infective therapy, and nutritional support. However, the patient’s condition continued to deteriorate with multiple organ failure, and the family opted to discontinue treatment.

### 2.5 Review of medical history

Since 2019, the patient had multiple hospital admissions due to pulmonary exudative lesions and improved after anti-infective therapy on each occasion. In October 2020, she was readmitted with pulmonary exudative lesions. A biopsy of the right upper lobe revealed granulomatous inflammation, and tuberculosis could not be ruled out. She subsequently received a 2HRZE/4HR anti-tuberculosis regimen: a 2-month intensive phase with daily oral Isoniazid, Rifampin, Pyrazinamide, and Ethambutol, followed by a 4-month continuation phase with daily oral Isoniazid and Rifampin. On May 26, 2023, she presented with ulcers on the left dorsum of the hand and the right lower leg. A skin biopsy confirmed granulomatous inflammation with microabscess formation, and tuberculosis could not be excluded. She was treated with a one-year oral R-EMfx-Clr regimen, consisting of daily Rifampin, Ethambutol, Moxifloxacin, and Clarithromycin.

**Figure 6:**
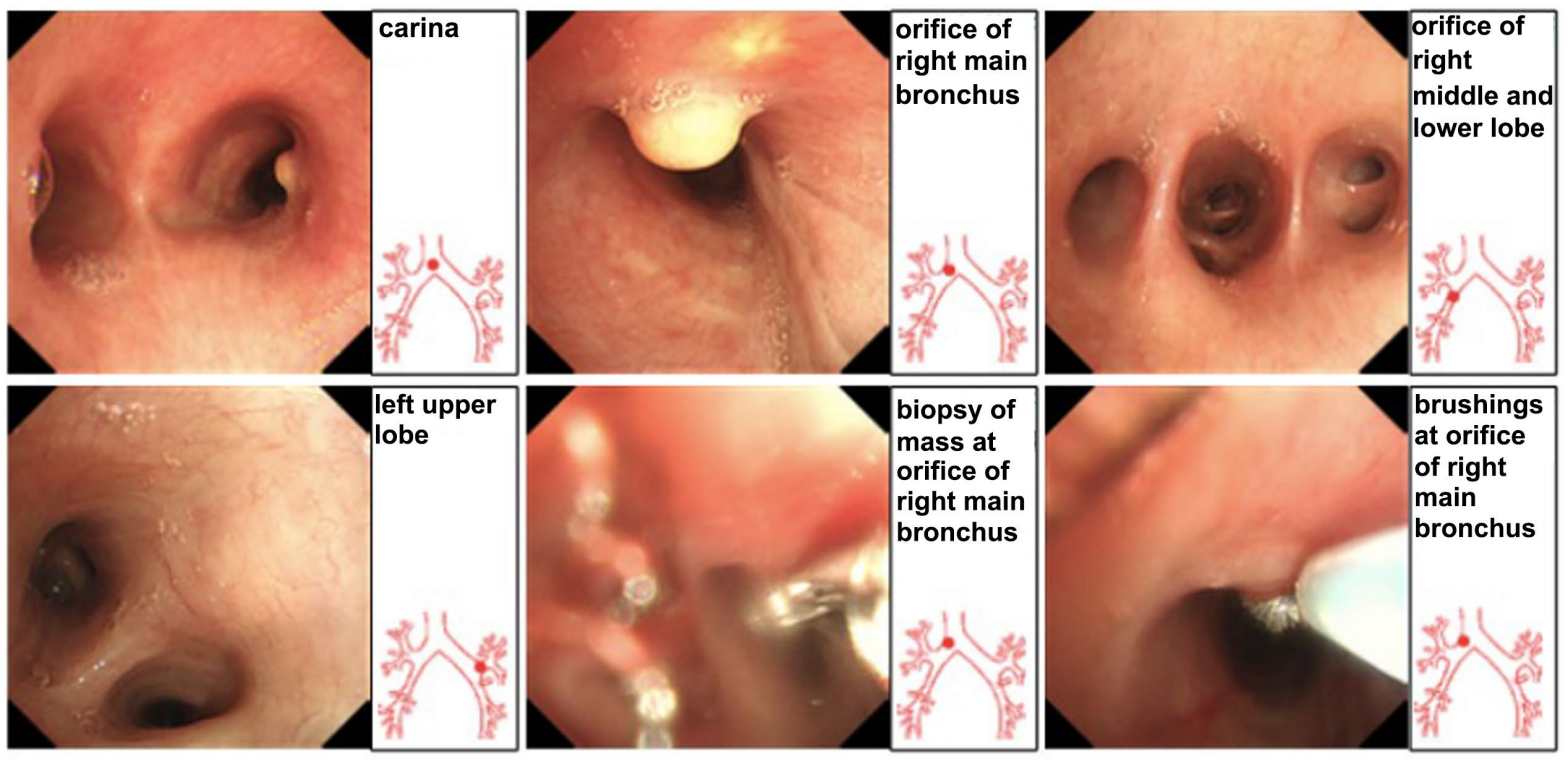
Bronchoscopy (August 19, 2024). No abnormalities in glottis, trachea, or carina. The lumens of both left and right bronchi showed no narrowing or deformation; mucosa smooth with no congestion or edema. At the 3 o’clock position of the right main bronchus opening, a yellow polypoid neoplasm protruding into the lumen was visible. Three biopsies obtained from the lesion. A brush biopsy was subsequently performed.

**Figure 7:**
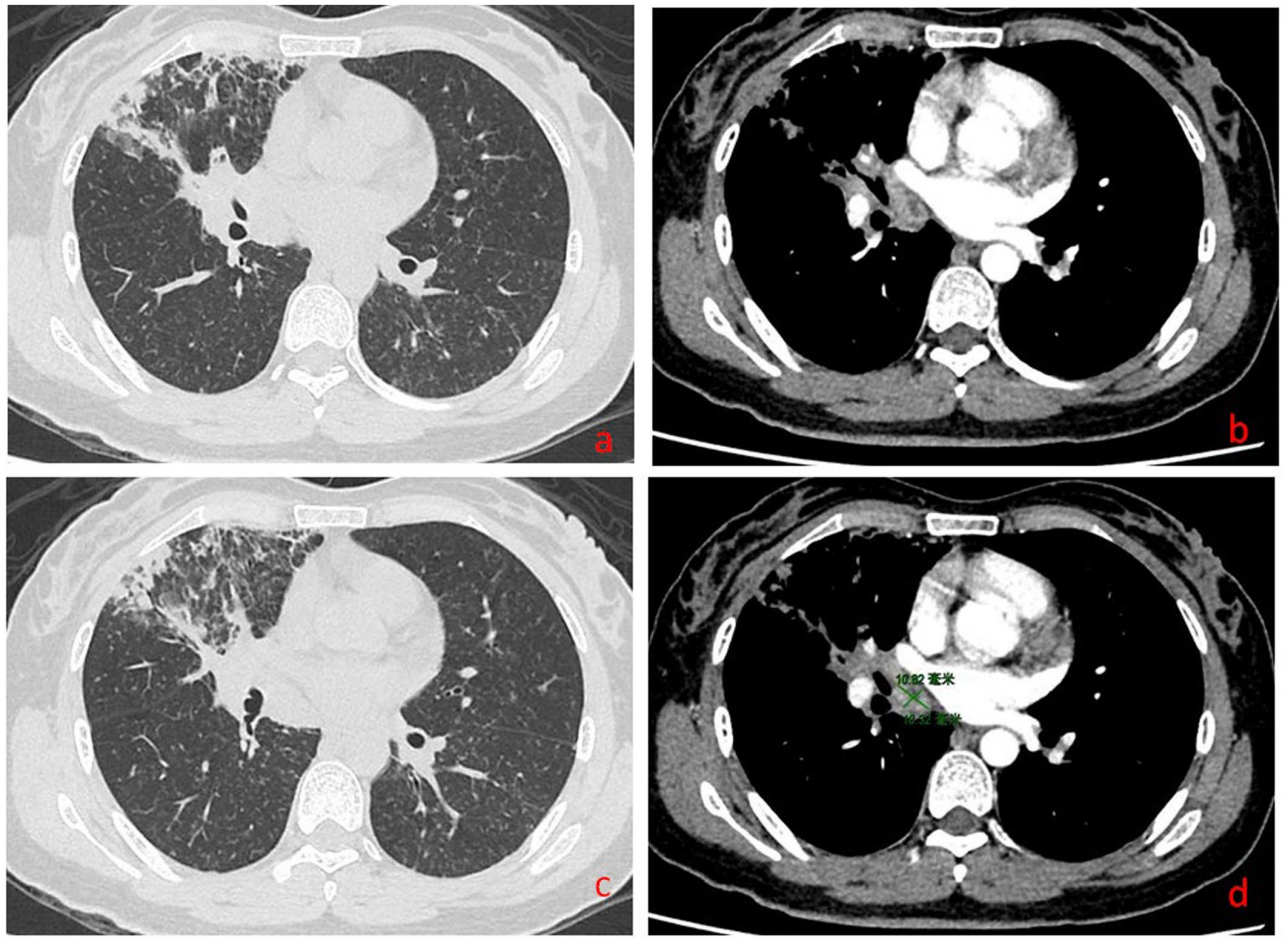
Chest CT (September 27, 2022). **(a-d)** Soft tissue density shadow adjacent to the right middle lobe hilum with corresponding bronchial narrowing. Contrast‑enhanced and angiographic imaging show abnormal enhancement and vascular compression. Multiple lesions and enlarged lymph nodes are seen in both lungs. Bronchoscopy confirmed *Aspergillus fumigatus* complex.

On August 19, 2024, bronchoscopy identified a new growth in the right main bronchus (Figure 6). A biopsy of this lesion confirmed granulomatous inflammation with abscess formation. During her hospitalizations, repeated cultures of both bronchoalveolar lavage fluid (BALF) and sputum grew *Aspergillus fumigatus* complex (Figure 7), but these findings were not adequately addressed. Considering the recurrent infections over the six-year period since 2019, characterized by shifting and migratory lesions, an anti-gamma interferon autoantibody test was performed on a blood sample to further investigate the etiology. The result was positive for anti-gamma interferon antibodies with a titer of 1:2,500.

## 3 Discussion

### 3.1 Clinical features and pathogenesis of AIGA syndrome

Adult immunodeficiency syndrome caused by anti-interferon-γ autoantibodies represents a distinct clinical syndrome characterized by disseminated non-tuberculous mycobacterial or other opportunistic infections, predominantly occurring in previously healthy adults (3, 5, 6). To date, over 800 cases have been reported globally, with Thailand and Taiwan accounting for two-thirds of these cases. Most patients reside in Southeast Asia or are of Asian descent. The pathogenesis of this syndrome is primarily associated with neutralizing antibodies blocking the interferon-gamma signaling pathway. Interferon-gamma is a key cytokine that activates macrophages and eliminates intracellular pathogens. Anti-interferon-gamma autoantibodies block its binding to receptors, thereby inhibiting STAT1 phosphorylation and suppressing the IL-12/IL-23-IFN-gamma positive feedback loop, ultimately impairing immune function (7–10).

This patient exhibited typical features of recurrent infections over 6 years. However, both the previously considered tuberculosis and non-tuberculous mycobacterial infections were only clinical diagnoses lacking microbiological or pathological confirmation. Following this hospitalization, the lesions in the middle lobe of the right lung increased compared to previous findings, further demonstrating the recurrent nature of the pulmonary infections. Simultaneously, the infection was widespread, involving multiple sites including the lungs, skin, pericardium (manifesting as pericardial effusion and constrictive pericarditis), and later developing abdominal wall abscesses and mediastinal infection, consistent with a pattern of multisite, disseminated infection. Pathological examinations repeatedly revealed granulomatous inflammation, consistent with chronic inflammatory responses triggered by immune dysfunction. Serum antibody testing ultimately revealed high-titer positive anti-interferon-γ autoantibodies (1:2,500). Peripheral blood lymphocyte subset analysis showed relatively low CD4+ T-cell counts and an inverted CD4+/CD8+ T-cell ratio, indicating impaired cellular immune function consistent with the typical clinical phenotype.

The patient had no clear family history, suggesting an acquired autoimmune mechanism as the predominant factor. Epidemiological studies indicate that anti-interferon-γ autoantibody-positive cases are relatively common in Southeast Asian populations, with their occurrence potentially associated with alleles such as HLA-DRB1*16:02 and HLA-DQB1*05:02 (11, 12). HLA typing was not performed in this case due to resource limitations and the retrospective nature of the diagnosis; however, the patient’s southern Chinese origin aligns with the endemic geographic distribution (13). This case emphasizes that in patients from Southeast Asia presenting with unexplained recurrent granulomatous infections, AIGA syndrome should be high in the differential diagnosis (Table 2).

**Table 2:** Serial laboratory trends.

Date	CRP (mg/L)	IL-6 (pg/mL)	Voriconazole level (μg/mL)	Clinical event
November 12, 2024	79.68	39.54	8.83	Admission
November 29, 2024	96.06	18.76	2.26	Post-pericardiectomy
December 19, 2024	91.99	124.86	6.38	Voriconazole level checked
January 3, 2025	55.74	42.18	Not done	Disease progression, switched to amphotericin B
January 23, 2025	93.10	78.65	2.22	Readmission with abscess

### 3.2 Microbiological characteristics of rare *Aspergillus* species

Interferon-γ is essential for antifungal immunity, activating macrophages and enhancing neutrophil killing of *Aspergillus* conidia and hyphae. Deficiency of this pathway due to neutralizing autoantibodies predisposes patients to invasive aspergillosis, particularly by low-virulence cryptic species that would otherwise be contained by intact cellular immunity.

The *Aspergillus udagawae* detected in the patient’s pericardial tissue via mNGS and the *Aspergillus siamensis* cultured from the chest wall drainage fluid both belong to rare species within the *Aspergillus* genus. Their unique biological characteristics may be key factors in the patient’s infection persisting over a prolonged period rather than progressing acutely. *Aspergillus udagawae* was first isolated and identified from Japanese soil in 1990. It is a close relative within the *Aspergillus* flavus complex of the *Aspergillus* genus. However, compared to common pathogenic *Aspergillus* species (such as *Aspergillus fumigatus* and *Aspergillus flavus*), it exhibits significant differences in growth characteristics and virulence (14).

This species differs markedly from common pathogenic *Aspergillus* species: First, it exhibits slow growth and weak spore production, with colony formation on culture media taking 2–3 days longer. This hinders rapid proliferation and acute inflammation in the early stages, contrasting with typical *Aspergillus* infections that worsen within 1–2 weeks. Second, its virulence is lower, with reduced production of virulence factors like fumonisins and weaker tissue invasion capacity. Acute-phase inflammatory markers (CRP, IL-6) in infected patients rise to only about half the levels seen in *A. fumigatus* infections. Severe complications like pulmonary necrosis or vascular invasion rarely occur in the short term, consistent with the patient’s early presentation of recurrent granulomatous inflammation without acute septic shock (15–19).

Notably, the *Aspergillus siamensis* species cultured from the patient’s chest wall drainage fluid is also a rare pathogen, commonly found in tropical regions and often grouped with species like *Aspergillus udagawae* under the classification of “cryptic species.” These fungi typically exhibit similar chronic infection characteristics, including slow growth, reduced virulence, and often diminished sensitivity to first-line antifungal drugs (such as voriconazole), complicating clinical management. Regarding drug susceptibility, *Aspergillus udagawae* demonstrates a higher minimum inhibitory concentration (MIC) for voriconazole than *Aspergillus fumigatus*, limiting the efficacy of standard voriconazole dosing (4, 20–22).

In this case, the patient’s lesions continued to proliferate despite initial treatment with voriconazole 200 mg twice daily. Symptoms improved only after switching to amphotericin B, which exhibited a lower MIC value against the pathogen, confirming this characteristic. The detection of *Siamaspergillus* in this mixed infection further suggests that such rare *Aspergillus* species may share similar drug-resistant phenotypes, leading to poor efficacy with initial voriconazole monotherapy (23, 24). In summary, the characteristics of “slow growth, low virulence, and unique drug susceptibility” exhibited by the *Aspergillus udagawae*/*Aspergillus siamensis* mixed infection, combined with the chronic immune deficiency caused by the patient’s anti-gamma interferon autoantibodies, formed a “low-virulence infection + weak immune response” pattern. This resulted in a long-term, protracted infection rather than the acute fulminant aspergillosis commonly seen in non-immunodeficient patients. This highlights the need for early molecular next-generation sequencing (mNGS) to identify fungal species in immunocompromised patients with chronic, slowly progressing, recurrent aspergillosis. Treatment regimens should be adjusted based on antimicrobial susceptibility testing to avoid delays.

### 3.3 Diagnostic challenges and role of mNGS

There are currently no unified diagnostic criteria for anti-interferon-γ autoantibody syndrome. Its clinical manifestations are highly diverse, bacterial culture sensitivity is low, and anti-interferon-γ autoantibody testing is not widely available, leading to frequent diagnostic delays. Early diagnosis of this syndrome is challenging but critically important. For adults without a history of known immunodeficiency who present with disseminated non-tuberculous mycobacterial or mycobacterial infections, complex infections involving multiple pathogens, or unexplained recurrent fever with multisystem involvement (e.g., lymph nodes, skin, bones) and abnormal immune markers-particularly those from southern China or Southeast Asia-serum anti-IFN-γ autoantibody testing should be performed (5, 25–27).

The pericardial involvement in this case closely mimicked tuberculous pericarditis, a common presentation in endemic regions. Table 3 summarizes key differentiating features between tuberculous and *Aspergillus* pericarditis. The availability of mNGS from pericardial tissue was crucial in identifying A. *udagawae* and avoiding prolonged unnecessary anti-tuberculosis therapy. This case underscores the value of molecular diagnostic methods in identifying rare pathogens in chronic granulomatous infections.

**Table 3:** Differential diagnosis: tuberculous versus *Aspergillus* pericarditis.

Feature	Tuberculous pericarditis	*Aspergillus* pericarditis (this case)
Epidemiology	Endemic in developing countries	Rare, associated with immunodeficiency
Clinical presentation	Subacute, constitutional symptoms, chest pain, dyspnea	Similar; indistinguishable clinically
Pericardial fluid	Lymphocytic exudate, elevated adenosine deaminase	Neutrophilic or mixed; ADA normal
Imaging	Pericardial thickening, effusion, sometimes calcification	Similar; may have associated pulmonary nodules
Microbiology	Acid-fast bacilli smear/culture, Xpert MTB/RIF	Fungal culture, galactomannan, mNGS
Pathology	Caseating granulomas with Langhans giant cells	Granulomatous inflammation with fungal hyphae
Response to therapy	Improves with anti-tuberculosis therapy	Progressive on anti-TB therapy; responds to antifungals
mNGS	*M. tuberculosis* sequences	*Aspergillus* species sequences

### 3.4 Treatment difficulties and drug resistance

For anti-interferon-γ autoantibody immunodeficiency syndrome, patients often fail to respond to antimicrobial therapy and experience frequent relapses due to the impact of gamma interferon autoantibodies on gamma interferon-induced downstream activities (8, 11, 22). Studies indicate that despite prolonged antimicrobial treatment with good compliance, opportunistic infections persist or recur, primarily due to the presence of high-titer autoantibodies in these patients (25, 28, 29). Therefore, prophylactic therapy should be initiated after completing standard antimicrobial treatment courses. Currently, no definitive effective treatment exists to reduce anti-gamma-interferon antibody titers. Although rituximab has been used as an adjunctive therapy in combination with antimicrobials in a small number of patients, it is costly and lacks long-term follow-up data (30). Cyclophosphamide has been reported to reduce autoantibody counts and lower serum anti-interferon-gamma antibody titers, with successful treatment outcomes in such patients, though it carries significant toxic side effects (31). Glucocorticoids or combination immunoglobulin therapy have also demonstrated varying efficacy in some cases (32). Immunomodulatory agents were withheld due to infection exacerbation risk, cost constraints, lack of protocols, and limited evidence.

In this case, the patient presented with invasive *Aspergillus* infection affecting both the pericardium and lungs. Treatment sequentially included voriconazole, isavuconazole, and amphotericin B. Although amphotericin B initially improved symptoms, the patient subsequently developed infections in other sites, leading to recurrent exacerbations and ultimately an unfavorable outcome. This demonstrates that current treatment strategies remain significantly limited when confronting such complex cases.

### 3.5 Novelty and literature review

A systematic literature search of PubMed, Web of Science, and CNKI databases (up to December 2025) using the terms “*Aspergillus udagawae*,” “pericardial aspergillosis,” “constrictive pericarditis,” and “anti-interferon-γ autoantibody” revealed no prior reports of pericardial infection caused by *A. udagawae*. To date, *A. udagawae* infections have been described primarily in pulmonary, cutaneous, and disseminated forms, predominantly in immunocompromised hosts. This case represents the first documented instance of pericardial involvement by this species.

The combination of pericardial and pulmonary involvement by two distinct rare *Aspergillus* species (*A. udagawae* and *A. siamensis*) in the setting of AIGA syndrome is also unprecedented. This case expands the clinical spectrum of aspergillosis and highlights the importance of species-level identification in guiding therapy, given the variable antifungal susceptibility profiles among cryptic *Aspergillus* species.

### 3.6 Implications for clinical practice and future research

The clinical and imaging manifestations of this case closely resemble those of pericardial tuberculosis, leading to initial diagnostic challenges and a high risk of misdiagnosis. This highlights that *Aspergillus udagawae* infection can mimic the classic features of tuberculosis. For “tuberculosis-like” cases unresponsive to conventional antimicrobial therapy, proactive pursuit of molecular microbiological evidence (e.g., mNGS) is essential to rule out fungal infection. Additionally, the reduced susceptibility of this strain to voriconazole further complicates treatment, underscoring the critical value of early, precise species identification and antifungal susceptibility testing in improving prognosis.

During treatment, only symptomatic therapy was administered, failing to reduce serum autoantibody titers—likely contributing to disease progression. In clinical practice, patients with recurrent infections and immune dysfunction should undergo early testing for anti-interferon-γ autoantibodies. Where feasible, immunomodulators (e.g., rituximab, cyclophosphamide, glucocorticoids) may be considered to lower antibody titers. Tailored treatment plans should be developed based on individual patient characteristics, including pathogen profile, immune status, and drug tolerance.

Currently, understanding of anti-interferon-γ antibody syndrome remains limited, and its rarity necessitates further research to elucidate pathogenesis and develop more effective therapies. Future efforts should focus on mechanisms of autoantibody production, improved detection methods, novel immunomodulatory regimens, and multidisciplinary management models.

## Data Availability

The original contributions presented in the study are included in the article/supplementary material, further inquiries can be directed to the corresponding author/s.
